# A review of health system infection control measures in developing countries: what can be learned to reduce maternal mortality

**DOI:** 10.1186/1744-8603-7-14

**Published:** 2011-05-19

**Authors:** Julia Hussein, Dileep V Mavalankar, Sheetal Sharma, Lucia D'Ambruoso

**Affiliations:** 1 University of Aberdeen, Foresterhill, Aberdeen, UK; 2 Indian Institute of Management, Vastrapur, Ahmedabad, India; 3 Bournemouth University, Bournemouth, UK

**Keywords:** maternal mortality, puerperal sepsis, infection control, nosocomial infections, health systems, developing countries

## Abstract

A functional health system is a necessary part of efforts to achieve maternal mortality reduction in developing countries. Puerperal sepsis is an infection contracted during childbirth and one of the commonest causes of maternal mortality in developing countries, despite the discovery of antibiotics over eighty years ago. Infections can be contracted during childbirth either in the community or in health facilities. Some developing countries have recently experienced increased use of health facilities for labour and delivery care and there is a possibility that this trend could lead to rising rates of puerperal sepsis. Drug and technological developments need to be combined with effective health system interventions to reduce infections, including puerperal sepsis. This article reviews health system infection control measures pertinent to labour and delivery units in developing country health facilities. Organisational improvements, training, surveillance and continuous quality improvement initiatives, used alone or in combination have been shown to decrease infection rates in some clinical settings. There is limited evidence available on effective infection control measures during labour and delivery and from low resource settings. A health systems approach is necessary to reduce maternal mortality and the occurrence of infections resulting from childbirth. Organisational and behavioural change underpins the success of infection control interventions. A global, targeted initiative could raise awareness of the need for improved infection control measures during childbirth.

## Introduction

The importance of a strong health system as the essential route to achieving improvements in maternal health and reductions in maternal mortality is widely accepted [1]. Effective coverage of maternity services requires timely and affordable access, by all sectors of the population, to appropriate care of sufficient quality and safety to help assure positive health outcomes. Good access, safety and quality are the overriding aims of all health systems and such factors are crucial when considering the problem of infections resulting from childbirth. Improving and maintaining infection control as part of delivery care requires an efficiently functioning health system.

Labour and delivery are especially hazardous times of pregnancy. Apart from the risks of severe bleeding and obstructed labour, life threatening infections can be introduced into the mother and baby's organs and bloodstream. 'Maternal sepsis' is a general term which has been used to include various obstetric and genito-urinary tract infections introduced into the mother [2]. The World Health Organization ranks maternal sepsis as the sixth leading cause of disease burden for women aged 15-44 years, after depression, HIV/AIDs, tuberculosis, abortion and schizophrenia. As many as 5.2 million new cases of maternal sepsis are thought to occur annually and an estimated 62,000 maternal deaths will result from the condition [2]. Added to the burden of loss of women's lives caused by sepsis are the long term consequences of infertility and the association of maternal sepsis with over one million infection related neonatal deaths every year [3,4].

A specific form of maternal sepsis is known as puerperal sepsis, an infection which is introduced during childbirth, but manifests in the post partum period within the first 42 days after delivery. It is of special importance because it is a serious, life threatening disease of the mother with infection of the womb and abdominal cavity, bloodstream infection, fever and pain [5]. In industrialized countries, puerperal sepsis is rare, causing 2.1% of maternal deaths. In Latin America and the Caribbean, its contribution to maternal mortality is 7.7%, ranking lower than hypertensive disorders, haemorrhage, obstructed labour and abortion. In Africa and Asia, it is the second commonest cause of maternal mortality after haemorrhage, causing 9.7% and 11.6% of deaths respectively [6]. Other infections resulting from childbirth cause a considerable burden of morbidity and include infections of the genital tract, Caesarean section wound infections and urinary tract infections, but are usually not life threatening.

In developing countries, many women still deliver at home, making prevention of infection at home and in the community important, especially if family members and traditional birth attendants are unaware of the need for infection prevention. The provision of delivery care by health professionals and in health facilities is expected, and indeed, likely to decrease infection rates because of use of clean practices, sterile gloves and instruments. Yet the tumultuous history of puerperal sepsis and its association with institutional delivery care and the birth attendant is well recorded. Infective organisms causing puerperal sepsis are often introduced when the birth attendant conducts invasive procedures such as vaginal examination, instrumental or caesarean delivery. When childbirth in hospitals became more common in Western countries in the early 20^th ^century, an increase in maternal mortality occurred, much of which was due to spread of infection between women in labour by the attending health professional and use of invasive obstetric procedures [7]. This occurred despite knowledge of how infections were spread which dated back to the mid 19^th ^century. Increasing concerns of hospital and healthcare associated infections are also currently recorded across many medical disciplines, even in high income, industrialised countries [8]. Given these experiences, the increasing use of health facilities for childbirth in developing countries [9] calls for an attitude of watchfulness. In India, for example, the national policy promotes institutional deliveries which have steadily increased in the last 15 years from 26% to 41% [10,11]. Studies here have shown that sepsis could be responsible for as much as 40% of maternal deaths [12,13]. In Mexico, 84% of deliveries occur in health facilities and rising Caesarean section rates were over 27% in the public sector and 70% in the private sector in 2005 [14]. Here, septic shock has been documented to account for as much as 5 to 10% of mortality [15]. There is no direct evidence of infection rates rising as a result of increasing institutional delivery rates. However, it is plausible that increasing utilisation of under resourced health facilities can result in stresses to the health system, overcrowding, poor environmental conditions, overworked health workers, shortages of drugs and supplies and sub standard clinical practices. These falling standards of care may include deteriorating infection control practices, resulting in an increased risk of institutionally acquired puerperal sepsis.

The epidemiology and aetiology of puerperal sepsis and other infections resulting from childbirth in developing countries are reviewed elsewhere [6,16-18]. Specific interventions necessary to prevent and treat infections are well known and include good hand hygiene, antisepsis, surgical sepsis and antibiotics. However, evidence on the more complex interventions relating to improvement of compliance, practice and behaviour is less well documented. Drawing from the broader infection control literature, this article reviews health system infection control measures pertinent to labour and delivery units in developing country health facilities.

## Methods

A structured literature review was conducted between March and May 2009. The objective of the review was to inform the development of strategies to prevent infections transmitted during labour and delivery in developing country health facilities. We searched for literature which described infection control measures. Evidence of effectiveness was of interest, but we did not restrict our review to these studies as we wished to ascertain whether ideas were being tried out that required further testing. We anticipated that infection control measures were likely to be implemented within wider health system activities, so we did not confine our search only to maternity care.

The review was structured in so far as a list of terms was defined and used to search electronic data bases in a methodological manner, but was not intended to be a systematic review with pre-defined data extraction forms, plans for data synthesis, quality assessment or specific selection criteria [19]. The electronic bibliographic databases MEDLINE, EMBASE, CINAHL, POPLINE and the Cochrane library were searched to 2009 with no earlier date or language restriction. The databases were searched using the following terms alone and in combination: infection control AND [mater* OR neonat* OR health care OR health system OR quality care] AND [sepsis OR infection] AND [control OR prevent*]. No language restrictions were applied. Over 2000 articles were initially found. The titles and abstract of the articles were screened. First, articles on infection control interventions in community settings, antenatal care, abortion care, interventions directed specifically at the child or neonate and on specific infections (e.g. malaria, tuberculosis, HIV) were excluded. In a second step, we attempted to find articles from developing countries and related to infection during childbirth and safe or clean delivery. Almost no relevant articles were identified, so we included articles of general infection control measures and from developed countries. This yielded 116 articles.

The abstracts of these 116 resulting articles were scrutinised by two of the authors independently and in some cases, full texts were retrieved with 54 articles eventually found to be relevant to our objective. The selection of these 54 articles was not based on specific criteria, but on subjective decision making based on whether the article provided useful information on potential means to prevent infection transmission in labour and delivery units and whether interventions described were likely to overcome problems and challenges relevant to health systems in low resource settings.

Websites were searched based on the authors pre-existing knowledge of international agencies working in relevant areas, including United Nations agencies (UNICEF, UNFPA, World Health Organization); maternal health groups (American College of Nurse Midwives, Engender Health, International Confederation of Midwives, International Federation of Gynaecologists and Obstetricians, JHPIEGO, John Snow Inc, Partnership for Maternal, Newborn and Child Health); and quality, patient safety and surveillance organisations (Agency for Healthcare Research and Quality, Centres for Communicable Diseases, National Institute for Health and Clinical Excellence, Patient Safety Alliance, Quality Assurance Project). Web based citations from published papers were also searched.

## Findings

The articles included had a global or overall developing country perspective. Reviews and guidelines were found (Table 1) and primary studies seeking to evaluate the effects of various infection interventions (Table 2). The developing countries included in the studies were in Asia (Nepal, India, Pakistan, Thailand), Africa (Egypt, Malawi, Mozambique, South Africa) and South America (Argentina and Columbia).

**Table 1:** Examples of infection control guidelines

Guideline	Focus	Description	References and weblinks
Infection Prevention Guidelines for Healthcare Facilities with Limited Resources (JHPIEGO)	General infection prevention	Tailored to low resource situations and for adaptation to the local setting. Targets education and behaviour change in both outpatient and hospitals settings and includes general medical, surgical, and obstetric services. It is one of a series of manuals, resource packages and videos on infection control. The manual covers 4 main areas: General infection prevention; processing of instruments; gloves and other items; implementing infection prevention in healthcare facilities; nosocomial infections.	Tietjen, Bossemeyer & McIntosh 2003 [58]http://www.reproline.jhu.edu/english/4morerh/4ip/IP_manual/ipmanual.htm

Practical Guidelines for Infection Control in Health Care (World Health Organization)	General infection prevention	Provides comprehensive information to health care workers on the prevention and control of transmissible infections. Builds on international guidelines and applies these to the needs of developing countries in Asia. Provides directions and information in relation to: Facilities, equipment, and procedures; cleaning, disinfecting and reprocessing of reusable equipment; waste management; protection of health care workers from transmissible infections; infection control practices in special situations.	World Health Organization 2004 [59]http://www.searo.who.int/LinkFiles/Publications_PracticalguidelinSEAROpub-41.pdf

Guide to the Implementation of the Multimodal Hand Hygiene Improvement Strategy (World Health Organization)	Hand hygiene	Targets health care facilities with all levels of resource availability. Concentrates on increasing compliance by health care workers. Main components: Improvement of infrastructure for hand hygiene; increase in knowledge and perception about hand hygiene, health care associated infection and patient safety.	WHO 2009 [60]http://www.who.int/entity/gpsc/5may/Guide_to_Implementation.pdf

Guideline for Hand Hygiene in Health Care Settings (Centres for Disease Control)	Hand hygiene	Provides health care workers with evidence and recommendations to promote improved hand hygiene practices and reduce infection transmission to patients and personnel. Describes physiological and pathological processes and defines key terms used in infection control. Reviews efficacy of various hand hygiene products and practices.	Boyce & Pittet 2002 [29]http://www.cdc.gov/hicpac/pubs.html

Guidelines for Environmental Infection Control in Health Care Facilities (Agency for Healthcare Research and Quality, USA)	Environment	Aims to provide evidence-based recommendations for environmental infection control in health-care facilities. The control measures are focused on prevention of infections associated with air, water, surfaces, laundry and bedding, medical wastes and animals of the environment. It is based on recommendations of the Centres for Disease Control and the Healthcare Infection Control Practices Advisory Committee in the USA.	http://www.guideline.gov/summary/summary.aspx?doc_id=3843&ss=15[61]

Clinical Guideline for Surgical Site Infection, (National Collaborating Centre for Women's and Children's Health and the National Institute for Health and Clinical Excellence, UK)	Surgical procedures	One of a series of infection control guidelines issued by NICE. D the prevention and treatment of surgical site infection except for specified specialised areas. The document reviews the evidence and provides recommendations for all procedures during the preoperative, intraoperative and postoperative phases of surgery.	NICE 2003 [62]http://www.nice.org.uk/nicemedia/pdf/CG2fullguidelineinfectioncontrol.pdf

**Table 2:** Studies on effectiveness of multifaceted infection control measures

Intervention	Focus	Setting	Design	Duration of intervention	Key findings	Reference
Issue of guidelines	Centres for Disease Control hand hygiene guidelines	40 hospitals, USA	Before and after, no control	2 years, with follow up for 1 year after release of guidelines	All hospitals changed policies, procedures and products after guideline introduced 90% staff were aware of guidelines No change in hand hygiene compliance	Larson et al 2007[36]

Education: Monthly meetings for feedback; posted infection rates in wards; voluntary educational group sessions; distribution of infection control manual	Hand hygiene	Intensive care units in one hospital, Argentina	Before and after, no control	21 months, with 16 month follow up after intervention	Hand washing compliance increased from 23% to 65% Infection rates decreased from 5 to 3 per 100 patient days	Rosenthal et al 2005 [37]

Organisational and systems improvements: Interactive development and placement of posters; distribution of alcohol based hand rub products; support from senior management	Hand hygiene, particularly alcohol based hand rubs	One hospital, Switzerland	Before and after, no control	3 year follow up after intervention	Consumption of alcohol hand rub by volume increased from 4 to 15 litres per 1000 patient days Hand hygiene compliance increased from 48% to 66% Infection rates decreased from 17% to 10%	Pittet et al 2000 [28]

Surveillance, including: Epidemiological analysis; prioritisation of infection during ward rounds; feedback to staff; specialised infection control staff; improved staff to bed ratios	Urinary tract, surgical, bacteremic infections and pneumonia	Representative sample of 4,000 hospitals, USA	Quasi-experiment-al, with regression modelling	5 years	A maximum decrease in infection rates by 32% if all components implemented Most hospitals could only achieve reductions in infection rates of 6% Different combinations of components were optimally effective for different infections	Haley et al 1980 [42] Haley et al 1985 [40]

Continuous quality improvement: Teamwork; analysis of cause-effect using problem based models; prioritisation of specific actions emerging from problem solving	Caesarean section	2 obstetric referral hospitals, Colombia	Segmented time series	2 years	Administration of antibiotic prophylaxis increased from 71% to 95% in hospital A and from 36% to 89% in hospital B Downward trend in surgical site infection rate in both hospitals	Weinberg et al 2001[45]

Characteristic problems related to infection control in developing countries include bad antibiotic prescribing practices, poorly functioning laboratory services, lack of surveillance data and sub-optimal design or construction of buildings and water and sanitation systems. Overcrowding of facilities and insufficient numbers of health workers are commonly noted. Increased bed numbers, nurse to patient ratios and bed space are known to have negative effects on infection transmission. Managers roles are not well specified, which contributes to the poor quality of services [20-23]. The combination of limited resources and general health conditions such as malnutrition, anaemia, and underlying infectious disease pose added risks [24]. Given such challenges, establishment of good infection control practices are believed to require a broad spectrum of interventions which address the availability and use of appropriate technologies, clear procedural guidelines and functionality of the health system [25-27].

Technological advances for preventing and treating puerperal sepsis in health facilities have been reviewed elsewhere and include supplies and equipment such as hand rubs and low cost disposable equipment, improved antibiotics and other drugs for treatment of severe infections, and microbiological diagnostic techniques [25,27]. Alcohol based antiseptic products are more efficacious than soap and water in reducing bacterial counts, and are convenient to use especially in basic health facilities where supplies of running water may be limited [21,28,29]. Systematic reviews have not however, found an established link between use of hand hygiene products and reductions in nosocomial infections [30]. The application of antiseptic washes to the vaginal area during labour has received much current interest, but there is insufficient evidence of its effectiveness in preventing maternal infection [27,31,32].

The World Health Organization's Global Patient Safety Challenge was set up to highlight the need for multimodal approaches to prevent health care associated infections alongside technological innovations [26,33]. Increasingly, multifaceted and multicomponent interventions, which draw from psychological, educational, organisational, administrative, technological and medical perspectives, are being evaluated [34]. Such interventions, which are implemented either alone or in combination, include guideline use, education and training, organisational change, surveillance and quality improvement.

### Guidelines

Various guidelines or procedural documents describing actions or recommended practices for infection control, for industralized and low resource settings have been issued. Examples are provided in Table 1. Some guidelines, such as those on hand hygiene, are highly specific and have been developed using quality assessed evidence meticulously gathered from reviews of literature. These have been the product of work done as part of the Global Patient Safety Challenge which targeted hand hygiene as a flagship campaign [24,35]. The effect of issuing new infection control guidelines specifically for promoting hand hygiene was evaluated across 40 hospitals in the USA [36]. No change in hand hygiene practices were found despite apparent uptake of the guidelines into hospital policies (Table 1). The lack of a comprehensive approach involving various levels within the organization, poor administrative support and absent feedback mechanisms were thought to have been reasons for the failure to change practice.

### Education

Educational interventions were categorised as those which improve skills or knowledge by training activities or by providing feedback on performance. In Argentina, an educational strategy which combined training sessions and performance feedback was used to improve hand hygiene in an intensive care unit. Focused, frequent education sessions were provided to health care workers. The education sessions emphasised the use of guidelines on hand hygiene and also fed back information to health workers on performance [37]. Hand washing compliance was observed covertly and infection rates improved markedly over the 16 months after the intervention was initially introduced (Table 2). Similar effects from education and performance feedback were noted in other settings in Argentina [38].

### Organisational and systems changes

Organisational and systems interventions were those that involved administrative, budgetary or management inputs, adjustments to staffing structures or roles and changes in policy or governance. A multimodal hand hygiene strategy comprising educational inputs, feedback and organisational change was evaluated in Switzerland [28]. The organisational interventions included ensuring that strong institutional support was developed by gaining involvement of clinical directors, obtaining funding from senior management budgets and ensuring that senior clinicians participated actively at meetings. Emphasis was placed on making individual bottles of hand rubs available and improving bedside access to hand hygiene products. The study showed improvements in hand washing compliance and infection rates (Table 2). Effects were followed up for over three years after the intervention package was introduced. Other studies have demonstrated similar effects but none for such a sustained period of time [29]. In Egypt, an organisational structure was set up to develop national guidelines, train and establish monitoring and evaluation systems, but no results on effectiveness of the programme were available [39].

Other organisational changes include reviewing health facility staffing and the way in which personnel are organised. These appear to be important aspects for success, along with professional infection control and clinical epidemiological expertise [40]. More recent studies have been conducted to establish the optimal knowledge and skills of infection prevention specialists and of staff-to-bed ratios, but clear recommendations on effective organisation of staff have not yet emerged [41].

### Surveillance

A national epidemic of nosocomial staphylococcal infection in American hospitals in the 1950s and 1960s prompted a number of efforts to assess the effects of surveillance, which involves systematic monitoring of events or performance. Several uncontrolled studies in the 1970s subsequently demonstrated its effectiveness in reducing infection rates, but it was the seminal SENIC (Study on the Efficacy of Nosocomial Infection Control) findings which are of greatest interest [40,42]. The study identified the extent to which hospitals were conducting surveillance and showed that surveillance, combined with other infection control activities, led to reductions in nosocomial urinary tract infection, surgical wound infection and bacteraemia. Prevention of up to a third of infections could be achieved if maximum intensity activities were undertaken, but few hospitals managed to implement all components (Table 2). The components included surveillance, feedback, training and adequate staffing to bed ratios. An infection control nurse working with specially trained physicians or microbiologists with special interests in infection control was required to supervise the programme. Routine identification of nosocomial infections during clinical ward rounds, analysis of rates of infection using epidemiologic techniques, and periodic use of data generated in decision-making were also important. The exact combination of components that seemed to be the most effective varied for the different sites of infection. Of particular interest to maternity care, prevention of bacteraemia, which is the main condition associated with life threatening puerperal sepsis, required what was termed the highest 'intensity' activities - involving most or all of the components [40].

### Continuous quality improvement

More recent studies have echoed the importance of multimodal, high intensity combinations. Real time reminders, provider audits, feedback and continuous quality improvement activities have been recommended [43]. Continuous quality improvement is a means of audit which follows a set process to create teamwork, identify problems and solutions and create shared goals using data for decision making [44]. A continuous quality improvement intervention was implemented in Colombia to improve infection rates after Caesarean section [45]. Surveillance systems and an infection control committee were set up. Multidisciplinary teams were formed. Individuals reviewed and summarised literature and discussed findings with team members as part of the educational process. The teams identified causes of infection relevant to their own context and developed realistic solutions according to the identified needs. The study found that prescribing practices for prophylactic antibiotic cover improved and infection rates dropped (Table 2). The cost investment for the intervention was reportedly modest, with activities conducted as part of routine clinical duties but specific data on time and monetary costs were not provided.

### Other infection control measures

Mandatory public reporting mechanisms for health care associated infections and the use of benchmarking to identify better and less well performing institutions have also been proposed [26,34,46]. The effect of introducing opinion leaders to motivate and change the practice of clinicians has been assessed in a systematic review [47]. Opinion leaders were more effective than feedback of information and didactic educational meetings, but these findings were relevant to improving the quality of maternity care in general, and were not specific to infection control.

### Cost-effectiveness

Cost-effectiveness data provide comparisons between the various costs and outcomes of two or more different interventions. The cost of extended hospitalisation due to infection is thought to exceed those of improving infection control measures. In the USA, reduction of infections by only 6% would offset the cost of an infection control programme by savings from reduced hospitalisation [40]. A systematic review of studies between 1990 and 2000, mostly from the USA, Canada and Europe, found that the costs attributable to bloodstream infections was the highest of different types of infection but lack of standardisation and methodological rigour of the studies constrained any conclusions [48]. A study in India showed that care for longer stay, hospital acquired bacteraemia in a cardiac hospital cost US$15,000 more per patient, when compared to patients who did not develop infection [49]. In Mozambique, single dose prophylactic antibiotics at emergency Caesarean section was found to cost less than a tenth of a post operative, seven day regimen, with no significant difference in infection rates [50].

## Discussion

The infection control measures described have been somewhat artificially categorised as it can be observed that each intervention, for example 'education' or 'surveillance' in fact comprises several other components such as performance feedback, use of guidelines or technological improvements. The least complex intervention found - introduction of new clinical guidelines - was not found to result in practice change [36]. To achieve optimal reductions in infection rates, there is some evidence that multimodal and multifaceted interventions are effective [40,43]. Most current studies suffer from the limitations of quasi-experimental designs, the lack of controls and the multi-component nature of the interventions. Evidence on cost-effectiveness in infection control is lacking. There are few evaluations on infection control measures in labour and delivery, yet the principles of infection control remain the same across clinical areas and in different resource settings, so the findings of the studies are relevant to maternity care. The link between infection control, the quality and safety of services and health system factors is widely recognised [21,29].

The findings of this review suggest a need to implement and evaluate complex and multifaceted approaches in obstetric units. The choice of the specific combination of components to be evaluated can be informed by what is known from the wider infection control literature, from existing information on ways to improve quality in maternity care and by tailoring strategies to address underlying problems of infection control [47]. Unnecessary or wasteful components need to be weeded out [21,50,51]. Measurement challenges remain and ways to standardise metrics across different settings, case mixes or facilities will allow better comparisons to be made between studies. Improvements of proxy indicators of morbidity, such as attributable length of stay have been called for [34] and may be especially relevant because puerperal sepsis is a comparatively rare event. For policy level decision making, the viability of allocating resources to infection control programmes would depend on demonstrating the merits between the costs of prevention and the costs of treatment [40]. The need for action in the implementation of infection control measures should not be seen as a competing priority with research support, especially where resources are limited. Studies of effectiveness should be designed to act as agents of change by also catalysing improvements in practice.

The interventions described in this review highlight the importance of behavioural change in the health workforce, yet such change is notoriously difficult to implement. Inadequate understanding of complex motivational factors may play a role [18]. Even if individuals are well motivated, working in a chaotic environment or a setting with poor infrastructure can be a barrier to change. Our knowledge of how specific characteristics or contexts affect behaviour is inadequate - for example, it is unclear why physicians and nursing assistants have been found to be poorer hand washers than nurses, or why compliance with infection control is more common during weekdays and in intensive care units [29]. Such uncertainties emphasise the need to study infection control interventions using a broad lens, combining knowledge from diverse perspectives such as psychology, education, organisational and management science and technology. Behavioural theories from models developed in psychology have been used to examine infection control practices such as hand washing in health care providers, concluding that it is the interdependence of various factors including environment, organisation and structure that matters, rather than individual behaviour [29]. Such understanding can help shape the design of appropriate interventions, so that instead of simplistic interventions targeting health workers to wash their hands, viable strategies are those that make changes which affect interactions between individuals, and how they function within their environment and their institutions. Recent expansion of patient safety initiatives in the developed world can be seen to draw from organisational, sociological and psychological theory [52,53], and will provide future lessons for infection prevention in developing countries and for maternity care.

As one of the leading causes of maternal mortality in low and middle income countries, it is surprising how little attention has been paid to puerperal sepsis and infection control during childbirth. Globalization of health policy and consequent responses can have varying impacts. On the one hand, some targeted approaches which focus on single causes of maternal mortality and morbidity in developing countries appear to have created momentum and interest for specific conditions. For instance, considerable attention has recently been given to studying the use of misoprostol and other technologies to improve the management of post partum haemorrhage in several low and middle income nations [27]. Scaled up, multicentre research has provided unequivocal evidence of the efficacy of magnesium sulphate in preventing deaths from eclampsia [54]. Advocacy for the condition is now part of the Clinton Global Initiative Commitment and an International Call to Action has been developed [55]. A global campaign to end obstetric fistula is supported by an array of international organisations in which obstetric care to prevent obstructed labour is a core activity [56]. On the other hand, a high profile, World Health Organization supported Global Patient Safety Challenge focusing on infection control, [26,33] has not resulted in actions to reduce infection risks in labour wards in developing countries.

An estimated 358,000 women die every year from the complications of childbirth and up to 15% of these are due to puerperal sepsis [57]. A simplistic extrapolation of the finding that infection rates can be reduced by 32% using optimal infection control measures [40], would suggest that the deaths of over 17,000 women could be prevented every year. Millions of women suffering from maternal sepsis and its long term consequences would also benefit. There should be no excuses for delaying targeted, global action to implement and evaluate infection control measures during labour and delivery for the prevention and reduction of puerperal sepsis and other related conditions.

## Conclusion

This review has highlighted three overarching lessons related to infection control and maternal mortality reduction. Firstly, despite limited evidence on effective infection control measures during labour and delivery and from low resource settings, it appears that education, surveillance, organisational change and quality improvement interventions should be introduced, confirming the need for a health systems approach to reduce maternal mortality, especially in relation to sepsis. Second is the need to improve our understanding of organisational and behavioural change to effectively implement infection control measures. In doing so, we will need to be informed by diverse and multidisciplinary perspectives. Finally, globalized, targeted health policies or initiatives have the potential to bring attention to, and catalyse action for what is currently a neglected, but important cause of maternal death worldwide.

## Competing interests

The authors declare that they have no competing interests.

## Authors' contributions

All authors were involved in reading drafts of the manuscript and providing comments and suggestions for the paper. They have all approved the final version of the paper. JH and DM provided guidance on the framework and direction of the literature review. JH wrote and redrafted manuscripts and reviewed articles. SS conducted literature searches, reviewed articles, prepared drafts of tables and drafted the methods section. LD reviewed articles and provided substantive inputs on drafts of the manuscript.
